# Genome-Wide Identification of the *CYP78A* Gene Family in *Lycium* and Functional Characterization of *LrCYP78A5*

**DOI:** 10.3390/plants14081152

**Published:** 2025-04-08

**Authors:** Yiru Zhao, Shupei Rao, Guoli Dai, Jinhuan Chen

**Affiliations:** 1 College of Biological Sciences and Technology, Beijing Forestry University, Beijing 100083, China; zhaoyiru@bjfu.edu.cn (Y.Z.); raoshupei1995@163.com (S.R.); 2 State Key Laboratory of Efficient Production of Forest Resources, Beijing Forestry University, Beijing 100083, China; 3 National Wolfberry Engineering Research Center, Ningxia Academy of Agriculture and Forestry Sciences, Yinchuan 750002, China; dgl2006swfc@163.com

**Keywords:** *Lycium*, *CYP78A* gene family, tissue specificity, genetic transformation, chlorophyll content

## Abstract

The *CYP78A* gene family, a plant-specific subfamily of cytochrome P450 enzymes, plays pivotal roles in plant growth, development, and stress responses. Although the *CYP78A* genes in many plants have been widely studied, little is known about them in *Lycium*. In this study, we identified six *CYP78A* genes in both *Lycium barbarum* and *Lycium ruthenicum* through comprehensive bioinformatics analysis. These genes exhibited high conservation in protein structure, gene organization, and conserved motifs. Phylogenetic analysis revealed they are close in terms of homology to *CYP78A* genes in *Arabidopsis*, tomato, and eggplant. *Cis*-acting element analysis of the promoter regions indicated that *CYP78A* genes are involved in light, hormone, and stress responses, with tissue-specific expression patterns observed across different developmental stages. Subcellular localization experiments confirmed that LrCYP78A5 is localized in the endoplasmic reticulum. Overexpression of *LrCYP78A5* in *L. ruthenicum* resulted in a significant increase in chlorophyll content, indicating the former’s potential role in plant growth. These findings provide valuable insights into the functional roles of the *CYP78A* gene family in goji, highlighting their potential involvement in growth regulation and metabolic processes.

## 1. Introduction

Cytochrome P450 (CYP) is an important superfamily of heme-containing proteins widely found in various organisms, including plants and animals [1,2]. In plants, these proteins play crucial roles in a wide range of biochemical pathways, including the biosynthesis of plant hormones such as auxins, which promote seedling growth [3]. The CYP78A family is a plant-specific subfamily of P450 enzymes, typically present in multiple copies within the genome [4]. *CYP78A5/KLUH* was the first member of the *CYP78A* gene family discovered in *Arabidopsis thaliana*. It was found to positively regulate cell size through cell proliferation, and overexpression of *CYP78A5/KLUH* in Arabidopsis resulted in larger organs, including leaves, seeds, and petals. Conversely, loss of *CYP78A5/KLUH* function led to smaller leaves and floral organs [5,6]. In addition to regulating organ size, *CYP78A5* also plays a role in plant stress responses. It enhances drought tolerance by regulating the synthesis of cuticular wax to reduce water loss and improves heat stress tolerance by reducing endoplasmic reticulum (ER) stress [7]. Recently, *AtKLUH* was also shown to positively regulate leaf longevity through cytokinin signaling and proline metabolism [8]. Furthermore, members of the *CYP78A* gene family may interact with the COPII complex, participating in intracellular vesicle transport and thereby regulating the transmission of growth signals [9]. In *Arabidopsis thaliana*, in addition to *CYP78A5,* there are five other members of the *CYP78A* subfamily, namely, *CYP78A6*, *CYP78A7*, *CYP78A9*, and *CYP78A10*. Among these, *CYP78A7* and *CYP78A5* have been found to play redundant roles in regulating the duration of the plastochrons, leaf size, and apical dominance [10,11]. *CYP78A6* (*EOD3*) and *CYP78A9* have been demonstrated to redundantly regulate seed size and leaf senescence, while *AtCYP78A8* is specifically implicated in the regulation of seed color without influencing leaf senescence [11,12,13]. The *CYP78A* gene family is primarily involved in plant growth and development, organ size regulation, stress responses, and metabolic regulation.

Goji is an eco-economic tree species distributed across Northwest China. It is an economically important plant used for both medicine and food, and its fruits are used in traditional Chinese medicine and as health foods [14,15]. In Tibetan medicine, *L. ruthenicum* is known as “Pangma” and has been used for centuries as a traditional remedy for fatigue caused by hypoxia [16,17]. Modern pharmacological studies have confirmed that black goji berry fruits are rich in anthocyanins, polysaccharides, vitamins, and eight essential amino acids, with pharmacological effects including improving vision, nourishing the liver and kidneys, lowering blood sugar levels, reducing the risk of cancer and cellular aging, and enhancing immunity [18].

In this study, the *CYP78A* gene family in *Lycium* was identified through bioinformatics methods. The gene structure, conserved motifs, cis-acting elements, chromosomal localization, and gene synteny relationships were analyzed. Based on transcriptome data, the expression patterns of the *CYP78A* genes in different tissues (leaves, buds, petals, pistils, seeds, sepals, stamens, and fruits at different developmental stages) were analyzed to determine whether the *CYP78A* family members exhibit tissue-specific expression. The *LrCYP78A5* gene was cloned from *L. ruthenicum*, and its subcellular localization was analyzed. Overexpression of *LrCYP78A5* in *L. ruthenicum* was induced through the leaf disc transformation method to explore the function of the *LrCYP78A5* gene, providing a theoretical basis for further research into the biological functions of the *CYP78A* gene family.

## 2. Results

### 2.1. Genome-Wide Identification and Characterization of CYP78A Genes in Goji

To identify *CYP78A* family members, the Blastp program was employed, and six *Arabidopsis* thaliana CYP78As were used as query proteins to screen the genomes of *Lycium ruthenicum* and *Lycium barbarum*. Following the initial screening, the hidden Markov model (HMM) of the P450 domain was downloaded from the Pfam database for further analysis. The candidate genes were subsequently evaluated using the NCBI-CDD and SMART databases to exclude those lacking the conserved P450 domain. Ultimately, six CYP78A genes were identified in each of the two genomes, *LbCYP78A5*-*LbCYP78A10* and *LrCYP78A5-LrCYP78A10*.

The physicochemical properties of the CYP78As identified were predicted based on their amino acid sequences. The protein lengths, molecular weights (MWs), isoelectric point (pI) values, instability index values, aliphatic index values, and predicted subcellular localization of the CYP78As are provided in Table 1.

Analysis of physicochemical properties revealed that the amino acid lengths of LbCYP78As range from 511 to 555 aa, with an average length of 527 aa. The molecular weights of these proteins range from 56.4 to 62.4 kDa, and their pI values range from 5.75 to 9.17. Notably, LbCYP78A6, LbCYP78A8, and LbCYP78A9 exhibit pI values greater than 7, suggesting that they may function as alkaline proteins. Similarly, the amino acid lengths of LrCYP78As range from 441 to 536 aa, with an average length of 510 aa. Their molecular weights range from 49.97 to 60.22 kDa, and their pI values range from 6.13 to 9.32. LrCYP78A6, LrCYP78A8, and LrCYP78A9 also display pI values greater than 7, indicating that they are likely alkaline proteins as well. Subcellular localization prediction showed that all *CYP78A* gene family members were localized in the endoplasmic reticulum, indicating that the CYP78A family members play roles in the endoplasmic reticulum. The *CYP78A* gene family members in *L. barbarum* and *L. ruthenicum* showed one-to-one homologous relationships, with CYP78A5, CYP78A6, and CYP78A10 having the same protein lengths in both species, suggesting functional conservation. CYP78A7, CYP78A8, and CYP78A9 had shorter protein lengths in *L. ruthenicum* compared to those in *L. barbarum*, possibly due to fragment deletion in *L. barbarum*.

### 2.2. Analysis of Phylogeny, Gene Structures, and Conserved Motifs of the CYP78A Family in Lycium

**Figure 1:**
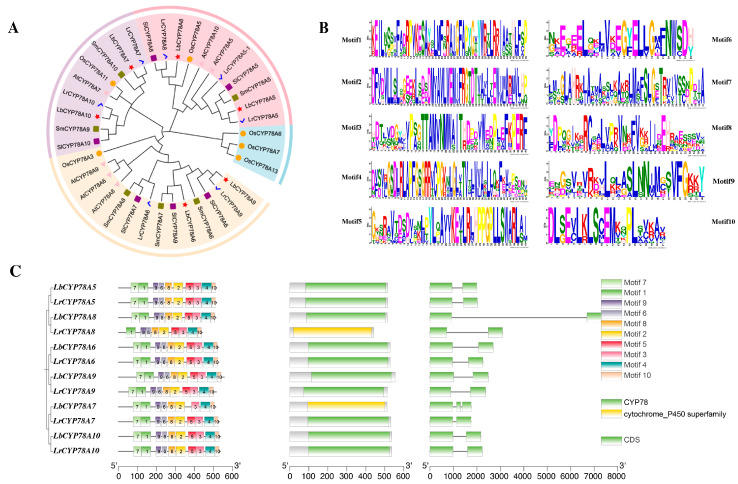
Phylogenetic relationships, gene structures, and conserved protein motifs of CYP78A genes in *Lycium.* (**A**) Phylogenetic tree of CYP78A protein family from *L. barbarum*, *L. ruthenicum*, *A. thaliana*, *S. lycopersicum*, and *S. melongena*. A maximum likelihood (ML) phylogenetic tree was constructed by using Tbtools with the Auto model and 1000 ultrafast bootstraps. (**B**) The motif logo of CYP78A proteins. (**C**) The motifs and gene structures of CYP78A proteins. The motifs, numbered from 1 to 10, are displayed in different colored boxes.

Gene structures and the conserved domains were further analyzed, and an ML phylogenetic tree was constructed using the identified CYP78As from different species. The analysis of phylogeny, motifs, and gene structures conducted using Tbtools-II v2.210 indicated similarities and differences between CYP78As. Based on the phylogenetic tree, these CYP78As proteins can be divided into four groups (Figure 1A), suggesting that CYP78As within the same group share common structural and functional characteristics. This allowed for the prediction of protein structures and function in *Lycium* species by referencing the well-studied CYP78As in model plants.

Domains are parts of a protein with specific functions and usually move or function independently of the rest of the protein [19]. Further analysis of the gene structures and conserved domains of *Lycium* CYP78As revealed that the members of the CYP78A family exhibit similar characteristics (Figure 1B). Among the six LbCYP78As, LbCYP78A7 contains two introns, and the others contain one. In contrast, all six LrCYP78A family members contain only one intron.

Ten conserved motifs that are shared among the 12 CYP78A proteins were identified using the MEME suite (Figure 1C). According to CDD analysis, all motifs except motif7 and motif1 belong to the CYP78 domain. Analysis of motif numbers revealed that the *Lycium* CYP78A gene family contains 9–10 motifs. Except for LrCYP78A8, which lacked motif7, the other 11 CYP78As contained 10 motifs in the same positions. Overall, the gene structures of the CYP78A family are relatively conserved, suggesting that CYP78A evolution is conserved across species.

### 2.3. Analysis of Cis-Acting Elements in the Promoter Regions of CYP78A Genes in Lycium

**Figure 2:**
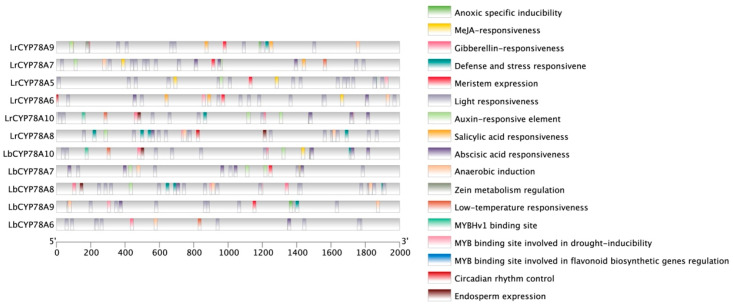
Prediction of *cis*-acting elements. *Cis*-element prediction for the 12 CYP78A gene promoter sequences (−2000 bp) was performed using PlantCARE technology. The figure shows 18 types of *cis-* elements.

To investigate the potential functions of *CYP78A* genes in *Lycium*, 2000 bp sequences upstream of the ATG translation start sites were extracted as promoter regions and analyzed using the PlantCARE online database (http://bioinformatics.psb.ugent.be/webtools/plantcare/html/, accessed on 2 April 2025). The analysis identified 18 types of *cis*-acting elements, primarily associated with light response, environmental stress response, and hormone response (Figure 2). Among the identified plant response elements, light response elements were the most abundant. Other environmental response elements included anaerobic induction, low-temperature response, and drought response elements. In addition to environmental response elements, the *CYP78A* genes also contained hormone-related response elements, such as gibberellin response, methyl jasmonate (MeJA) response, salicylic acid response, abscisic acid response, and auxin response elements.

**Figure 3:**
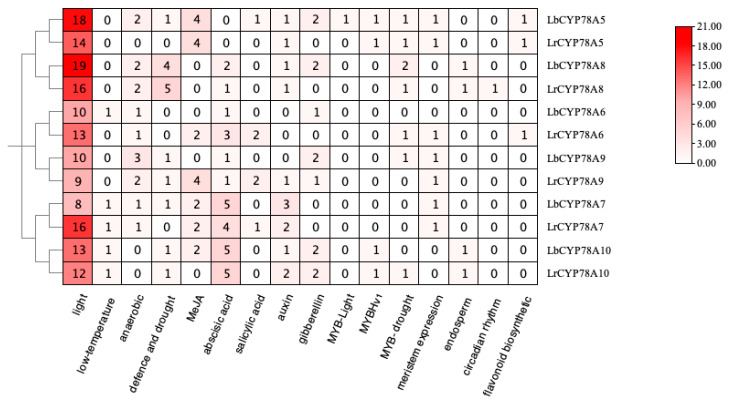
Clustered heatmap of the number of different elements. Darker colors indicate a larger number of specific elements in CYP78As.

The types and distribution of *cis*-acting elements in the promoter regions of *CYP78A* genes varied significantly. According to the clustered heatmap of *cis*-acting elements (Figure 3), light response elements were distributed across all 12 *CYP78A* genes (100%). In addition to light response elements, anaerobic induction and abscisic acid response elements were also abundant. Anaerobic induction elements were found in all *CYP78A* genes except *LbCYP78A5*, *LbCYP78A10*, and *LrCYP78A10*. Abscisic acid response elements were found in all *CYP78A* genes except *LbCYP78A5* and *LrCYP78A5*. Elements related to endosperm expression were only found in *CYP78A8* and *CYP78A10*, suggesting that these genes may be involved in endosperm development. Our analysis of *cis*-acting elements indicates that *CYP78A* genes in *Lycium* are widely involved in growth and development, hormone signaling, and stress responses.

### 2.4. Analysis of Covariance Between L. barbarum and Other Plant Species

**Figure 4:**
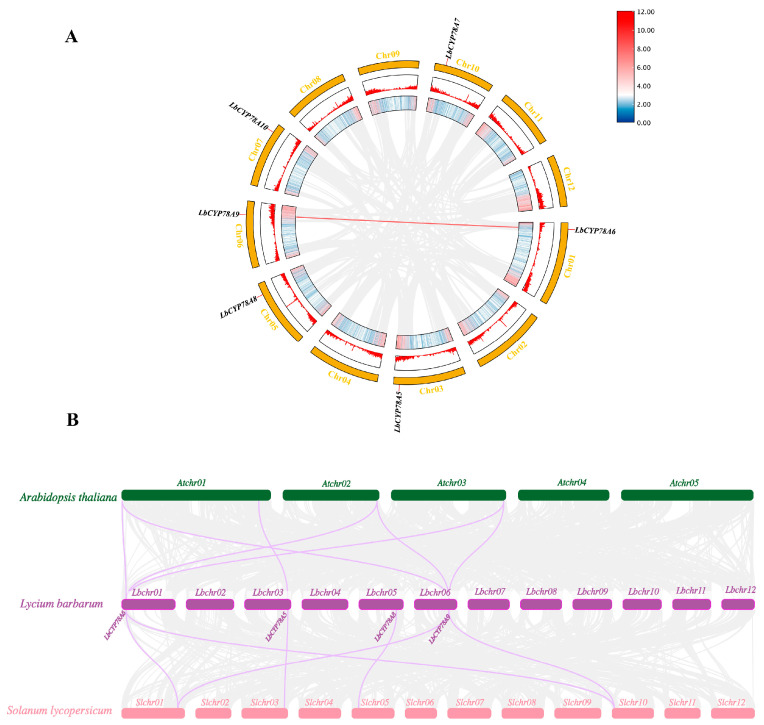
Covariance analysis of *CYP78A* genes: (**A**) intraspecies synteny analysis of *L.barbarum* and (**B**) interspecies synteny relationships between *L.barbarum*, *A. thaliana*, and *S. lycopersicum*.

The chromosomal localization results showed that the six *CYP78A* genes in *L. barbarum* were located on six chromosomes: Chr1, Chr3, Chr5, Chr6, Chr7, and Chr10 (Figure 4A). All six genes were located at the ends of the chromosomes, a phenomenon also observed in the genomes of eggplant, *Arabidopsis*, and tomato, suggesting a potential evolutionary conservation of gene positioning in these species. To identify tandem gene duplications in the *L. barbarum* genome, synteny analysis was performed using Tbtools, revealing that LbCYP78A6 and LbCYP78A9 were tandemly duplicated. These results suggest that this tandem duplication may contribute to the expansion of the CYP78A gene family in *Lycium*.

Comparative synteny analysis between *L. barbarum*, *A. thaliana*, and *S. lycopersicum*. revealed that *LbCYP78A5*, *LbCYP78A6*, *LbCYP78A8*, *LbCYP78A9*, and *LbCYP78A10* showed synteny with AtCYP78As and SlCYP78As (Figure 4B), indicating that these homologous genes evolved early and are functionally conserved.

### 2.5. Expression Patterns of CYP78A Genes in L. barbarum, and L. ruthenicum

**Figure 5:**
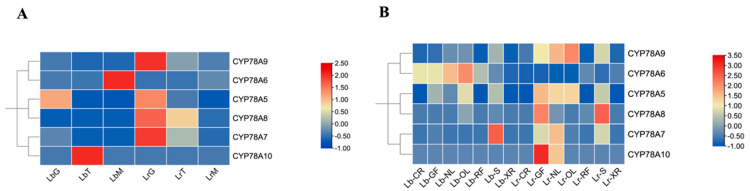
Expression pattern clustered heatmap of CYP78As. (**A**) clustered heat map of CYP78A expression patterns of *L. barbarum* (Lb) and *L. ruthenicum* (Lr) fruit at the green stage (G), turning-color stage (T), and mature stage (M); (**B**) clustered CYP78As expression patterns in stamens (XR), pistils (CR), green fruit (GF), mature fruit (MF), leaves on current-year branches (NL), leaves on last-year branches (OL), and stem tips (Ss) of *L. barbarum* (Lb) and *L. ruthenicum* (Lr).

To investigate whether *CYP78A* genes exhibit tissue-specific expression in *Lycium*, transcriptome data were used to analyze their expression patterns in *L. ruthenicum* and *L. barbarum* at different developmental stages (at the green fruit, color change, and mature fruit stages) and in different tissues (pistils, green fruits, new leaves, old leaves, mature fruits, stems, and stamens). The results showed that the six *CYP78A* genes exhibited significant differences in expression across different developmental stages (Figure 5A) and tissues (Figure 5B). *CYP78A6* was exclusively expressed during the mature fruit stage, while *CYP78A10* was specifically expressed during the color change stage in *L. barbarum.* In contrast, *CYP78A5* was exclusively expressed during the green fruit stage in both *L. barbarum* and *L. ruthenicum*, suggesting functional specificity of *CYP78A5* in the process of fruit ripening. Analysis of expression patterns in different tissues showed that *LbCYP78A10* was not expressed in any of the tissues of *L.barbarum*, but it was expressed in new and old leaves of *L. ruthenicum*. *LbCYP78A6* was only expressed in various tissues of *L.barbarum*, but not in *L. ruthenicum*, indicating functional differences between *LbCYP78A6* and *LbCYP78A10* in the two species.

### 2.6. Subcellular Localization of CYP78A5

**Figure 6:**
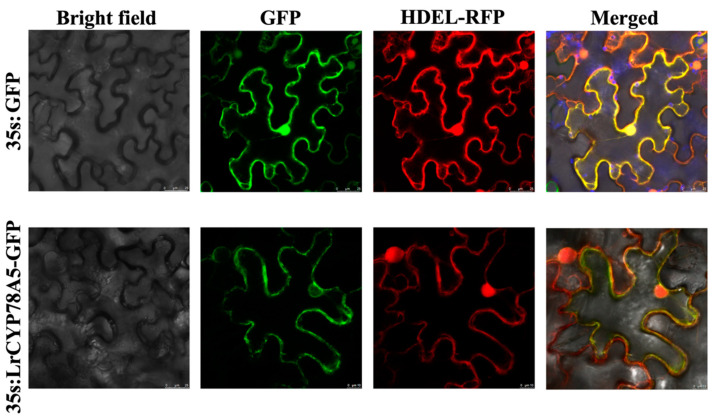
Subcellular localization of LrCYP78A5.

Based on the results in Table 1, subcellular localization prediction showed that all CYP78A family members in *Lycium* were localized in the endoplasmic reticulum. According to the results of the expression pattern and function prediction, we selected CYP78A5 for subcellular localization analysis. Subcellular localization showed that, driven by the 35S promoter, the LrCYP78A5 protein that fused to the EGFP protein was targeted toward the endoplasmic reticulum (Figure 6). This indicates that LrCYP78A5 is a membrane protein localized in the endoplasmic reticulum, suggesting that it functions in the endoplasmic reticulum.

### 2.7. Overexpression and Identification of LrCYP78A5 in L. ruthenicum

**Figure 7:**
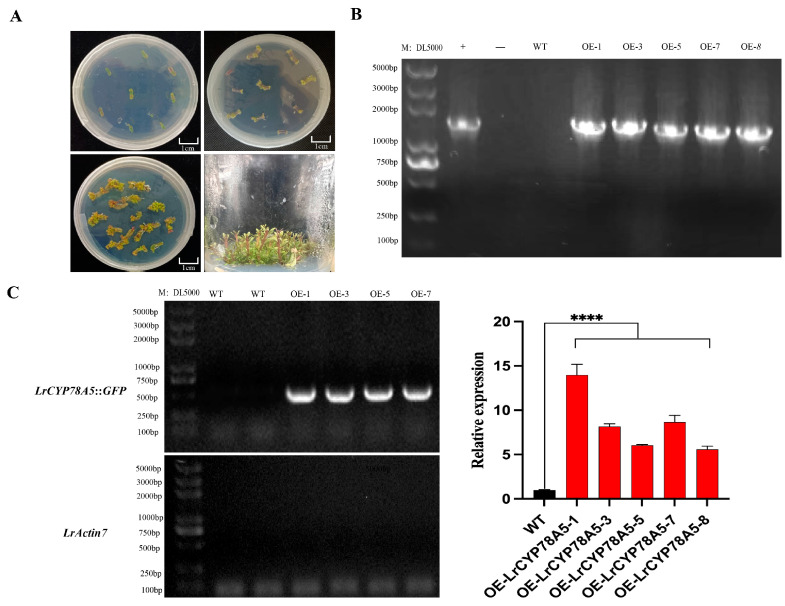
Genetic transformation process and RNA detection of *L. ruthenicum*. (**A**) The genetic transformation process for *L. ruthenicum*. (**B**) DNA detection of transgenic plants. (**C**) RNA detection of transgenic plants. According to Dunnett’s test, **** represent significant difference between WT and transgenic plants at *p* < 0.0001.

Based on the subcellular localization results, a genetic transformation of *CYP78A5* was performed to study its function. The *LrCYP78A5* transformation procedure in *L. ruthenicum* is shown in Figure 7A. Transgenic plants were validated through PCR-based identification and quantitative reverse transcription PCR (qRT-PCR) analysis. Five independent transgenic lines exhibiting high expression levels of the target gene were subsequently selected for further functional characterization.

### 2.8. Phenotypic Observation and Chlorophyll Content Measurement of LrCYP78A5-Overexpressing Plants

**Figure 8:**
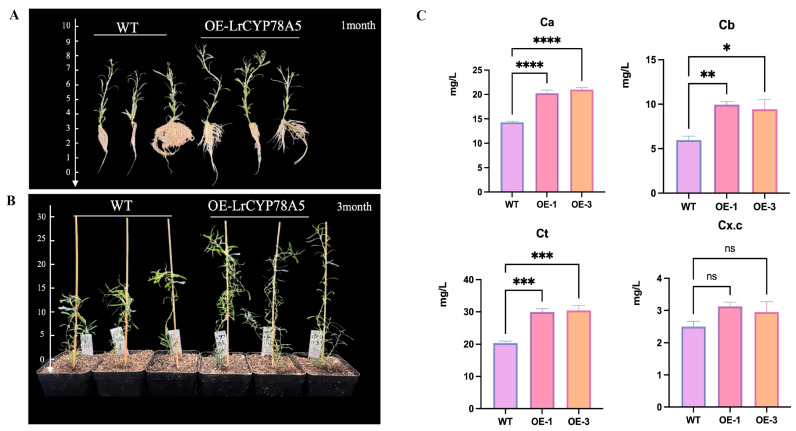
Phenotypic observations and chlorophyll content measurements for *LrCYP78A5*-overexpressing plants. (**A**) Phenotypic observation of one month of growth of wild-type and transgenic *L. ruthenicum*; (**B**) results of the observation of three-month phenotypes of *L. ruthenicum* and transgenic *L. ruthenicum* growth; (**C**) chlorophyll content measurement. Ca: total chlorophyll content; Cb: chlorophyll A; Ct: chlorophyll B; Cx.c: carotenoids.

Phenotypic observation of the transgenic plants (Figure 8A,B) showed that the transgenic plants grew faster than the wild-type plants after one and three months of growth. The transgenic plants appeared greener than the wild-type, consistent with the significantly higher chlorophyll content observed in the transgenic plants. Chlorophyll content measurement showed that the total chlorophyll content was significantly higher in the transgenic plants (Figure 8C), as were chlorophyll A (Chl a) and chlorophyll B (Chl b) levels. However, carotenoid content did not change significantly.

## 3. Discussion

In previous studies, it was demonstrated that the *CYP78A* gene family, widely present in plants, plays crucial roles in regulating organ size, stress responses, and metabolic regulation [20,21,22,23,24,25,26]. This study identified six members of the *CYP78A* gene family in *L. ruthenicum* and *L. barbarum*, exhibiting high conservation in terms of protein structure, gene structure, and conserved motifs.

Phylogenetic analysis revealed high sequence homology between *CYP78A* genes in *Lycium* species (goji berry) and those in *Arabidopsis thaliana*, *Solanum lycopersicum* (tomato), and *Solanum melongena* (eggplant). Notably, *CYP78A5*, *CYP78A6*, and *CYP78A10* exhibited identical protein lengths in both *L. ruthenicum* and *L. barbarum*, supporting the notion of functional conservation within this clade. Additionally, *CYP78A7*, *CYP78A8*, and *CYP78A9* showed protein fragment deletions in *L. ruthenicum* when compared to *L. barbarum*, possibly indicating gene fragment loss or functional divergence during evolution. These results indicate that the CYP78A family is highly conserved in *Lycium*. Furthermore, extensive research across various plant species, including rice, tomato, and maize, has consistently demonstrated the highly conserved functions of CYP78As within these plants [20,27,28].

Mechanistically, studies on *Arabidopsis* have established that *AtKLUH* (*CYP78A5*) regulates organ size non-cell-autonomously via the synthesis of a mobile signaling molecule [5,6,11]. Accumulating evidence suggests that this mobile signal may be derived from fatty-acid-related molecules [6,29,30]. In contrast, research on maize, wheat, and rapeseed has implicated CYP78As in auxin metabolism [31]. Consequently, further direct evidence is required to identify the specific mobile signal produced by CYP78As.

*Cis*-acting element analysis revealed that the promoter regions of the *CYP78A* gene family in *Lycium* contain abundant light response, hormone response, and stress response elements, suggesting that these genes may be widely involved in photosynthesis, hormone signaling, and stress responses in goji berry plants. Light response elements were present in all *CYP78A* genes, indicating their potential role in regulating photosynthesis. Some elements were only present in specific genes, suggesting functional specificity. For example, *CYP78A8* and *CYP78A10* showed high expression in the endosperm, indicating their potential involvement in seed development and maturation. The widespread distribution of abscisic acid and anaerobic induction response elements further suggests that the *CYP78A* gene family may be involved in drought and hypoxia stress responses in goji berry plants. Gene expression analysis showed that the *CYP78A* genes in goji berry plants exhibit significant differences in expression across different tissues and developmental stages. Some genes were highly expressed in specific tissues or developmental stages, while others showed low or no expression, indicating the tissue-specific and developmental-stage-specific functions of the members of the *CYP78A* gene family.

By cloning the *LrCYP78A5* gene and constructing an overexpression vector, we successfully obtained transgenic plants. Subcellular localization experiments showed that the LrCYP78A5 protein is localized in the endoplasmic reticulum, consistent with the subcellular localization predictions for the CYP78A family and the localization of AtCYP78A5 and TaCYP78A5 [5,32]. This further supports the hypothesis that *CYP78A* genes have functions in the endoplasmic reticulum and that the *CYP78A* family is highly conserved.

Leaves are the primary organs of photosynthesis and are essential for plant growth and development [33]. Chlorophyll is a key pigment for photosynthesis, and its content directly affects photosynthetic efficiency and plant growth. We found that the chlorophyll content in overexpressing *LrCYP78A5* was significantly higher than that in wild-type plants, suggesting that *LrCYP78A5* may regulate chlorophyll synthesis or stability to enhance photosynthetic efficiency in *Lycium*. This result aligns with the potential role of the *CYP78A* gene family in regulating photosynthesis and further suggests the high importance of *CYP78A* genes in plant growth and development.

## 4. Materials and Methods

### 4.1. Plant Materials

Three-year-old *L. barbarum* and *L. ruthenicum* seedling clones were cultivated at 38°38′51′′ N, 106°9′29′′ E. Stamens (XR), pistils (CR), pistils, green fruit (GF), mature fruit (MF), leaves on current-year branches (NL), leaves on last-year branches (OL), and stem tips (Ss) were collected. Green and red (mature) goji fruits were collected with three biological replicates from three different trees under normal cultivation and management conditions. Upon collection, all samples were promptly frozen in liquid nitrogen and subsequently preserved at −80 °C until the time of testing. To explore the genetic transformation system, four-week-old clonal *L. ruthenicum* plants stored in our laboratory were used. The clonal plants were cultured at 24 ± 1 °C under a 16 h photoperiod using cool-white fluorescent light with 3 klx intensity in a tissue culture room.

### 4.2. Identification of Members of the CYP78A Gene Family in Lycium

The whole-genome data for *Lycium* were downloaded from the goji berry plant database (https://figshare.com/articles/dataset/Wolfberry_genomes_and_the_evolution_of_Lycium_Solanaceae_/20416593, accessed on 1 January 2025) to identify members of the *CYP78A* gene family. Sequences of CYP78A proteins from *Arabidopsis* were obtained from the *Arabidopsis* Information Resource (TAIR) (https://www.arabidopsis.org). Potential CYP78A proteins were sought by querying the AtCYP78A protein sequence with the Auto Blast Two Sequences Set (E-value = 1 × 10^−5)^ in TBtools, and the hidden Markov model (HMM) of the CYP78A5 protein domain (ID: PF00067) was used to search against the *Lycium* genome in TBtools [34]. The candidate protein sequences were validated for protein domains using the SMART and NCBI-CDD databases, and sequences without complete protein domains were removed to confirm the members of the *CYP78A* gene family.

### 4.3. Analysis of CYP78A Proteins in Goji Berry

Basic physicochemical properties of the CYP78A proteins in *Lycium* were predicted using the ExPASy-ProtParam (https://ca.expasy.org) [35], and subcellular localization was predicted using Cell-Ploc-2 (http://www.csbio.sjtu.edu.cn/bioinf/Cell-PLoc-2/, accessed on 10 January 2025). The chromosomal locations of the *CYP78A* genes in *Lycium* were confirmed and visualized using the gene annotation file and Tbtools software.

### 4.4. Conserved Motif and Phylogenetic Analysis of CYP78A in Lycium

Conserved motifs in the CYP78A proteins of goji berry plants were analyzed using the MEME Suite 5.5.7 suite (https://meme-suite.org/meme/tools/meme, accessed on 17 January 2025), with the number of motifs set to 10. Gene structure analysis was performed using Tbtools, and the results were combined with the conserved motif analysis for visualization. Multiple-sequence alignment of CYP78A proteins from goji berry plants, Arabidopsis, tomato, and eggplant was performed using MEGA7.0 software, and a phylogenetic tree was constructed using the neighbor-joining method, with a bootstrap value of 1000.

### 4.5. Prediction of Cis-Acting Elements in the Promoter Regions of CYP78A Genes in Goji Berry

Using the PlantCARE database (http://bioinformatics.psb.ugent.be/webtools/plantcare/html/, accessed on 23 January 2025), promoters of 2000 bp sequences upstream of the CYP78A genes were analyzed to find possible *cis*-acting elements. Unnamed and non-functional *cis*-elements were removed, and the remaining elements related to plant growth and development, hormone regulation, and stress responses were retained. The results were visualized using Tbtools.

### 4.6. Expression Patterns of CYP78A Genes

The RNA-seq data corresponding to the fruit developmental stages of *L. barbarum* and *L. ruthenicum* (green fruit, color transition, and ripening stages) were retrieved from the NCBI Sequence Read Archive (SRA) under the accession number PRJNA483521 [36]. The FPKM (fragments per kilobase of exon model per million mapped fragments) values were calculated to quantify gene expression levels, and heatmaps were subsequently generated using Tbtools. Additionally, transcriptome data from various tissues, including new leaves from current-year branches (NL), old leaves from previous-year branches (OL), stems, pistils, stamens, green fruits, and ripe fruits of both *Lycium* species, were obtained from prior studies. Gene expression levels were determined using both FPKM and TPM (transcripts per kilobase of exon model per million mapped reads) metrics to ensure robust and accurate quantification.

### 4.7. Overexpression of LrCYP78A5 in L. ruthenicum

To investigate the functional role of *LrCYP78A5*, the full-length cDNA sequence was amplified using specifically designed primers. The purified PCR product of *LrCYP78A5* was subsequently fused to the N-terminal of green fluorescent protein (GFP) within the *Xba* I/*Sma* I-digested pBI121 vector, resulting in the construction of the recombinant plasmid pBI121-*LrCYP78A5*: GFP. This construct was then introduced into *Agrobacterium tumefaciens* strain GV3101 via the freeze–thaw transformation method, as previously described [37].

To achieve stable genetic transformation, the recombinant vector pBI121-*LrCYP78A5*: GFP was introduced into *L. ruthenicum* via *Agrobacterium*-mediated transformation. Initially, primary cultures of *Agrobacterium* tumefaciens harboring the transformation plasmids were grown in 5 mL of YEB liquid medium supplemented with 10 mg/L of rifampicin and 50 mg/L of kanamycin and incubated at 28 °C in the dark. Subsequently, the cultures were subcultured in 100 mL of YEB medium under identical conditions until the optical density at 600 nm (OD600) reached 0.6 to 0.8. The bacterial cells were then harvested and resuspended in MS medium to adjust the OD600 of the suspension to 0.5. Mature leaf explants from the fourth to sixth leaves of 28-day-old *L. ruthenicum* seedlings were excised and immersed in the *Agrobacterium* suspension for 5 to 10 min [38]. Following infection, the treated explants were placed in the medium and cultivated in the dark in an indoor environment for 2 days. Before being placed on the selected regeneration medium, the treated explants were rinsed three times with sterile water. Subsequently, the explants were transferred to selection medium, consisting of MS medium supplemented with 0.5 mg/L of 6-benzylaminopurine (6-BA), 0.1 mg/L of naphthaleneacetic acid (NAA), 300 mg/L of cefotaxime, and 30 mg/L of kanamycin, and incubated for 3 weeks. Regenerated shoots exhibiting kanamycin resistance were further screened by allowing them to root on a half-strength MS medium containing 0.1 mg/L of 3-indolebutyric acid (IBA).

### 4.8. Obtaining and Identifying Overexpression LrCYP78A5 Plants

Transgenic resistant tissue culture seedlings of black goji berry were obtained by preparing explants, inducing calluses, shoots, and rooting and screening for resistant plants. To identify the transgenic *L. ruthenicum*, the presence of T-DNA was first confirmed. When the resistant tissue culture seedlings developed into complete small plants, their leaves were collected for DNA detection. Genomic DNA was extracted from black goji berry leaf tissue using a Tiangen Plant Genomic DNA Kit (Sigma Aldrich, St. Louis, MO, USA), and PCR detection was performed. The PCR reaction system consisted of 20 μL:1 μL of DNA, 1 μL of forward primer (10 μmol/L), 1 μL of reverse primer (10 μmol/L), 10 μL of PrimeSTAR MAX premix (Takara, Beijing, China), and sterile distilled water to make up 20 μL. The PCR program was as follows: pre-denaturation at 98 °C for 3 min, followed by 34 cycles of denaturation at 98 °C for 10 s, annealing at 55 °C for 10 s, and extension at 72 °C for 30 s. The primers for *LrCYP78A5: GFP* were used to verify the successful transformation of *LrCYP78A5* in transgenic plants by confirming its integration into the plant genome. The transgenic plants were then subjected to qPCR analysis to examine the RNA transcription.

### 4.9. Subcellular Localization of LrCYP78A5

The pBI121-*LrCYP78A5: GFP* plasmid was transiently transformed in 28-day-old tobacco leaves using *Agrobacterium*-mediated transformation. The bacterial suspension was adjusted to an OD600 of 0.6–0.8 using MS medium containing 10 mmol/L of MgCl_2_, 10 mmol/L of MES, and 200 μmol/L of AS (Acetosyringone). After infection, the tobacco plants were grown in the dark for 1 day, with normal light cultivation for 2 days, and then imaged using a TCS SP8 X confocal microscope (Leica, Wetzlar, Germany), using a 488 nm laser line to image EGFP and chloroplast signals.

### 4.10. Real-Time Quantitative PCR Analysis

Total RNA was extracted from the leaves of *L. ruthenicum* using the Trizol method, and cDNA was synthesized using a reverse transcription kit, which was used according to the manufacturer’s instructions. RT-qPCR was performed using the TB Green Premix Ex Taq™ (Takara, Beijing, China) kit via a QuantStudio 6 Real-Time PCR System. The relative expression levels of transgenic *L. ruthenicum* were measured using *Actin7* as the internal reference gene. The reaction system and program were employed according to the manufacturer’s instructions. The specific primers are listed in Supplementary Table S1. The relative expression levels were calculated using the 2^−ΔΔCt^ method. Three biological replicates were used.

### 4.11. Determination of Photosynthetic Pigments

When the transgenic plants and the control group reached 45 days of growth, leaves from the middle section were collected and cut into small pieces approximately 2 mm in width. For each sample, 0.1 g of leaf tissue (with three biological replicates) was placed into a test tube containing 10 mL of extraction solution (acetone/absolute ethanol/water = 4.5:4.5:1). The tubes were sealed and stored in the dark for 32 h at room temperature to allow for complete pigment extraction. After extraction, the absorbance of the solution was measured at wavelengths of 663 nm, 645 nm, and 470 nm using a spectrophotometer. The concentrations of chlorophyll a, chlorophyll b, and carotenoids were then calculated according to the Arnon formula. This formula is given below:

Ca=12.21A663−2.59A646

Cb=20.13A646−5.03A663

CT=Ca+Cb

$$Cx.c=\frac{1000A470-3.27Ca-104Cb}{229}$$

Note: Ca and Cb represent the concentrations of chlorophyll a and chlorophyll b; Cx.c represents the total concentration of carotenoids; A663, A646, and A470 represent the absorbance of the chlorophyll extract at wavelengths of 663 nm, 646 nm, and 470 nm, respectively.

## 5. Conclusions

In this study, we conducted a comprehensive identification and functional characterization of the *CYP78A* gene family in *L. barbarum* and *L. ruthenicum*. Through bioinformatics analysis, six *CYP78A* genes were identified in each species, which revealed high conservation in terms of protein structure, gene organization, and conserved motifs. Phylogenetic analysis demonstrated close homology to *CYP78A* genes in model plants such as *Arabidopsis*, tomato, and eggplant, suggesting evolutionary conservation and functional redundancy. The analysis of *cis*-acting elements in the promoter regions highlighted the involvement of *CYP78A* genes in light, hormone, and stress responses, with tissue-specific and developmental-stage-specific expression patterns observed. Notably, in both species, *CYP78A6* and *CYP78A10* exhibited distinct expression profiles, indicating potential functional divergence. Subcellular localization confirmed that LrCYP78A5 is localized in the endoplasmic reticulum, consistent with its predicted role in cellular signaling and metabolite biosynthesis. Overexpression of *LrCYP78A5* in *L. ruthenicum* resulted in a significant increase in chlorophyll content, which suggests its role in enhancing photo-synthetic efficiency and plant growth. These findings underscore the importance of the *CYP78A* gene family in regulating growth, development, and stress responses in *Lycium*. The identification of *LrCYP78A5* as a key regulator of chlorophyll content provides a promising target for genetic improvement strategies aimed at enhancing crop productivity and stress tolerance. This study advances our understanding of the molecular mechanisms of the CYP78As underlying plant growth and stress responses. It also offers valuable insights useful for the genetic enhancement of *Lycium* and other economically important crops. Future research should focus on elucidating the specific molecular pathways regulated by *CYP78A* genes as well as exploring their potential applications in crop breeding and biotechnology.

## Figures and Tables

**Table 1:** Summary information of CYP78A family.

Gene Name	Gene ID	Amino Acid	Protein Molecular	Isoelectirc Point (pI)	Instability Index	Aliphatic Index	Predicted Subcellular Localization
		Length/aa	Mass/kDa				
LbCYP78A5	Lba03g02369	515	58.12	6.69	30.08	94.45	Endoplasmic reticulum.
LbCYP78A6	Lba01g00553	527	59.5	8.94	24.98	94.5	Endoplasmic reticulum.
LbCYP78A7	Lba10g00932	511	56.4	5.75	35.26	97.1	Endoplasmic reticulum.
LbCYP78A8	Lba05g01623	512	58.3	9.17	43.62	94.63	Endoplasmic reticulum.
LbCYP78A9	Lba06g01865	555	62.4	8.81	31.98	93.41	Endoplasmic reticulum.
LbCYP78A10	Lba07g01446	536	60.2	6.8	32.08	94.07	Endoplasmic reticulum.
LrCYP78A5	Lru01G036233	515	58.25	6.93	37.6	93.88	Endoplasmic reticulum.
LrCYP78A6	Lru01G045721	527	60.51	8.2	34.82	92.05	Endoplasmic reticulum.
LrCYP78A7	Lru01G004383	531	58.74	6.13	33.42	96.93	Endoplasmic reticulum.
LrCYP78A8	Lru01G025311	441	49.97	8.76	44.46	96.83	Endoplasmic reticulum.
LrCYP78A9	Lru01G034235	514	57.94	9.32	32.77	90.45	Endoplasmic reticulum.
LrCYP78A10	Lru01G043957	536	60.22	7.69	32.62	94.61	Endoplasmic reticulum.

## Data Availability

The transcriptome raw sequence data have been submitted to the NCBI Sequence Read Archive database under accession bioproject number PRJNA812857. The full cDNA sequences of *LrCYP78A5* and *LrActin7* have been submitted to GenBank under accession numbers PV368566 and PV368567, respectively.

## Supplementary Materials

The following supporting information can be downloaded at https://www.mdpi.com/article/10.3390/plants14081152/s1. Table S1: Primer sequences for RT-qPCR.
