# Supplementary material for: Genome-Wide Identification of the CYP78A Gene Family in Lycium and Functional Characterization of LrCYP78A5
Source: Plants (Basel). 2025 Apr 8;14(8):1152. doi: 10.3390/plants14081152 (PMC12030099; doi:10.3390/plants14081152)
Supplement: Supplementary file 1 [file plants-14-01152-s001.zip › plants-3551206-supplementary.pdf]

Supplementary Material

Table S1. Primer sequences for RT-qPCR

**Primer name**	**Primer sequence（5′- 3′）**
LrActin-F	CTGACGGTGAGGACATTCA
LrActin-R	GAGCATCATCTCCAGCAAAG
LrCYP78A5-F	TCTTGCCAGGATGGTTCTGC
LrCYP78A5-R	CGGAGCGAGCCTAAGATCAG
LrCYP78A5::GFP-F	TCTTGCCAGGATGGTTCTGC
LrCYP78A5::GFP-R	CGACACGCTGAACTTGTGG
